# Fisheries management in the face of uncertainty: Designing time-area closures that are effective under multiple spatial patterns of fishing effort displacement in an estuarine gill net fishery

**DOI:** 10.1371/journal.pone.0211103

**Published:** 2019-01-18

**Authors:** Liza A. Hoos, Jeffrey A. Buckel, Jacob B. Boyd, Michael S. Loeffler, Laura M. Lee

**Affiliations:** 1 Department of Applied Ecology, Center for Marine Sciences and Technology, North Carolina State University, Morehead City, North Carolina, United States of America; 2 North Carolina Division of Marine Fisheries, North Carolina Department of Environmental Quality, Morehead City, North Carolina, United States of America; 3 North Carolina Division of Marine Fisheries, North Carolina Department of Environmental Quality, Elizabeth City, North Carolina, United States of America; Swansea University, UNITED KINGDOM

## Abstract

A commonly cited reason for the failure of time-area closures to achieve fisheries management goals is the displacement of fishing effort from inside the closure into the surrounding area still open to fishing. Designing time-area closures that are predicted to achieve management goals under multiple spatial patterns of effort redistribution will increase chances of success. Using data from an estuarine gill net fishery, we tested if there are time-area closures predicted to reduce bycatch of two protected species groups while maintaining target catch under four simulated effort redistribution patterns. We found that the pattern of effort redistribution had a substantial impact on the amount of predicted bycatch in each closure scenario. Multiple closures were predicted to reduce bycatch of these species under all four simulations of effort redistribution. However, some combinations of closure and effort redistribution pattern resulted in estimated bycatch being higher than without a closure. We did not find any time-area closures that resulted in a predicted reduction in bycatch while maintaining target catch at original levels. We demonstrate a simple way for fisheries managers to account for the uncertainty in fishers' behavior by designing time-area closures that are predicted to reduce bycatch under multiple potential patterns of spatial redistribution of fishing effort.

## Introduction

Bycatch, or the unintentional catch of non-target species (e.g. finfish, sea turtles, marine mammals), is a global problem that occurs with almost every type of fishing gear [1]. Bycatch can contribute to population decline of the bycatch species and impact the larger ecosystem by removing or weakening key nodes of the food-web [2, 3]. Bycatch may also negatively impact fishers when bycatch mitigation regulations limit opportunities to fish for their target species or require costly gear modifications or replacement [3–5]. Ideally, approaches can be identified that reduce bycatch without negatively impacting the fishery’s profitability [6]. Providing incentives to reduce bycatch (e.g. quotas on bycatch that trigger fishery shutdown) have been shown to reduce bycatch rate but can require 100% observer coverage [7] which is cost-prohibitive for many fisheries. If finding such win-win solutions is not possible, an alternative objective is to find a solution that provides an optimal balance between a) the impact of bycatch on the health of the bycatch species’ population and ecosystem and b) the impact of bycatch mitigation strategies on the fishery [8].

One bycatch mitigation strategy that is used to strike a balance between conservation and fishery objectives is to implement time-area closures where high rates of bycatch occur, while still allowing the fishery to continue outside of the closure [2, 8]. Time-area closures are distinctive from permanent area closures and no-take marine reserves because they are implemented only temporarily or seasonally and often only prohibit a single gear type rather than prohibiting fishing entirely [9]. In practice, time-area closures for bycatch reduction have achieved mixed results, with both successes [10] and failures [11]. One commonly cited reason for failure is the displacement of fishing effort from the closure into the surrounding area still open to fishing [11, 12]. Depending on the spatial and temporal distribution of the bycatch, increased levels of effort outside of the closure due to displacement could lead to increased levels of bycatch, undermining or completely nullifying any bycatch reduction achieved by the removal of effort within the closure [13, 14].

Therefore, fisheries managers should not only consider the amount of bycatch reduction that would result from the cessation of fishing within a closure area, but also where displaced fishing effort may go and how it could potentially impact catch rates outside the closure [14–16]. However, fishers’ spatial response to a closure is difficult to predict as it will result from the sum of personal cost/benefit analyses made by each displaced fisher. The fishers’ response is based on a complex interaction of factors such as the current market rate, spatial distribution of their target species, specific fishing practices, the cost of fuel, the distance he/she is willing to travel, where he/she has made successful catches in the past, availability of alternative work, and weather conditions [4]. Observational studies of effort redistribution after a closure have documented a wide variety of spatial patterns that result from this decision-making process [17–19]. Given the difficulty in predicting fishers’ spatial response to a closure with certainty, one potential solution is choosing time-area closures that are predicted to achieve management goals under multiple spatial patterns of effort redistribution.

In this study, we test if there are time-area closures that reduce bycatch and maintain target catch under multiple possible effort redistribution patterns. We use data from a North Carolina (NC) estuarine gill net fishery that interacts with two protected species groups, sea turtles (loggerhead turtle (*Caretta caretta*), green turtle (*Chelonia mydas*), leatherback turtle (*Dermochelys coriacea*), hawksbill turtle (*Eretmochelys imbricate*), Kemp’s ridley turtle (*Lepidochelys kempii*)) and Atlantic sturgeon (*Acipenser oxyrinchus oxyrinchus*), to conduct this test and identify other issues that managers should consider when designing a time-area closure to reduce bycatch.

## Background

Large mesh gill nets are used by commercial fishermen throughout NC’s estuarine waters, which together compose the third largest estuarine system in the world [20]. Depending on the season and location, these fishermen may be targeting a wide variety of species, including southern flounder (*Paralichthys lethostigma*) and American shad (*Alosa sapidissima*). Large mesh gill nets can have high rates of bycatch [21] and capture protected species such as Atlantic sturgeon [20, 22] and several species of sea turtles [8, 23, 24] that migrate in and out of the estuary system throughout their life history. Bycatch in gill nets has been listed as one of the major sources of human-induced mortality for Atlantic sturgeon in the southeastern United States [22], and Atlantic sturgeon are listed as endangered in the Carolina Distinct Population Segment under the Endangered Species Act (ESA). Bycatch in fishing gear has also been cited as contributing to decline of sea turtle populations worldwide [25], and all five species of sea turtles that are found in NC waters are listed as endangered or threatened under the ESA. Of these five species, three are commonly caught in gill nets in NC [24]: the green sea turtle (listed as threatened in the North Atlantic Ocean Distinct Population Segment), loggerhead sea turtle (listed as threatened in the Northwest Atlantic Ocean Distinct Population Segment), and Kemp’s ridley sea turtle (listed as endangered throughout its range).

Under the ESA, it is illegal to capture (or “take”) any endangered or threatened species in fishing gear. Due to the possibility of unintended takes of these ESA-listed species, the NC Division of Marine Fisheries (NCDMF) must apply for an Incidental Take Permit in order for the large mesh estuarine gill net fishery to legally continue to operate. NCDMF estimates the number of sea turtle and Atlantic sturgeon takes on a daily, weekly, and monthly basis from data collected by fisheries observers. If the number of estimated Atlantic sturgeon or sea turtle takes is approaching the allowable limit for that management unit and time period (as specified in the Incidental Take Permit), large mesh gill nets will be prohibited for the remainder of that time period. Therefore, it is in both the fishers’ and fisheries managers’ best interest to reduce bycatch of these species. Although allocations of bycatch limits to individual fishers would be a larger incentive to reduce bycatch, this approach has not been used partly because of the large expense to have 100% observer coverage.

NCDMF uses a variety of strategies to ensure that the authorized number of takes is not surpassed [20]. These include gear and effort restrictions, mandated fishing seasons, and time-area closures. In addition to time-area closures that recur every year, NCDMF has divided NC’s estuary system into management units which can be closed to gill net fishing if sea turtle or Atlantic sturgeon takes are approaching the authorized number in a given time period. NCDMF may also delineate smaller closures within the management units in order to decrease the economic impact of a closure. Until this study, the delineation of these closures has been based primarily on previous spatial and temporal distribution of bycatch and did not explicitly take into account how redistribution of fishing effort in response to a closure might alter the expected outcome. We test if time-area closures can be identified that reduce bycatch of protected species in the large mesh gill net fishery under multiple possible effort redistribution patterns in two different fisheries: an American shad fishery in Albemarle Sound and a southern flounder fishery in Pamlico Sound.

### Atlantic sturgeon bycatch in Albemarle Sound

The majority of Atlantic sturgeon takes in the large mesh (>4 inches) gill net fishery in NC occurs in Albemarle Sound [20]. American shad and Atlantic Sturgeon are both anadromous species, spending a portion of their lives at sea and migrating into coastal rivers and tributaries to spawn [26]. Both of these species pass through the Albemarle sound on their way to spawn in the Roanoke and Chowan Rivers. American shad enter the Albemarle Sound in February and begin to exit in April [26], whereas Atlantic sturgeon enter the sound in the spring and remain through into the fall [20]. In the spring, the number of Atlantic sturgeon takes increase as the distribution of Atlantic sturgeon overlaps with the American shad fishery in the western portion of the sound.

**Fig 1:**
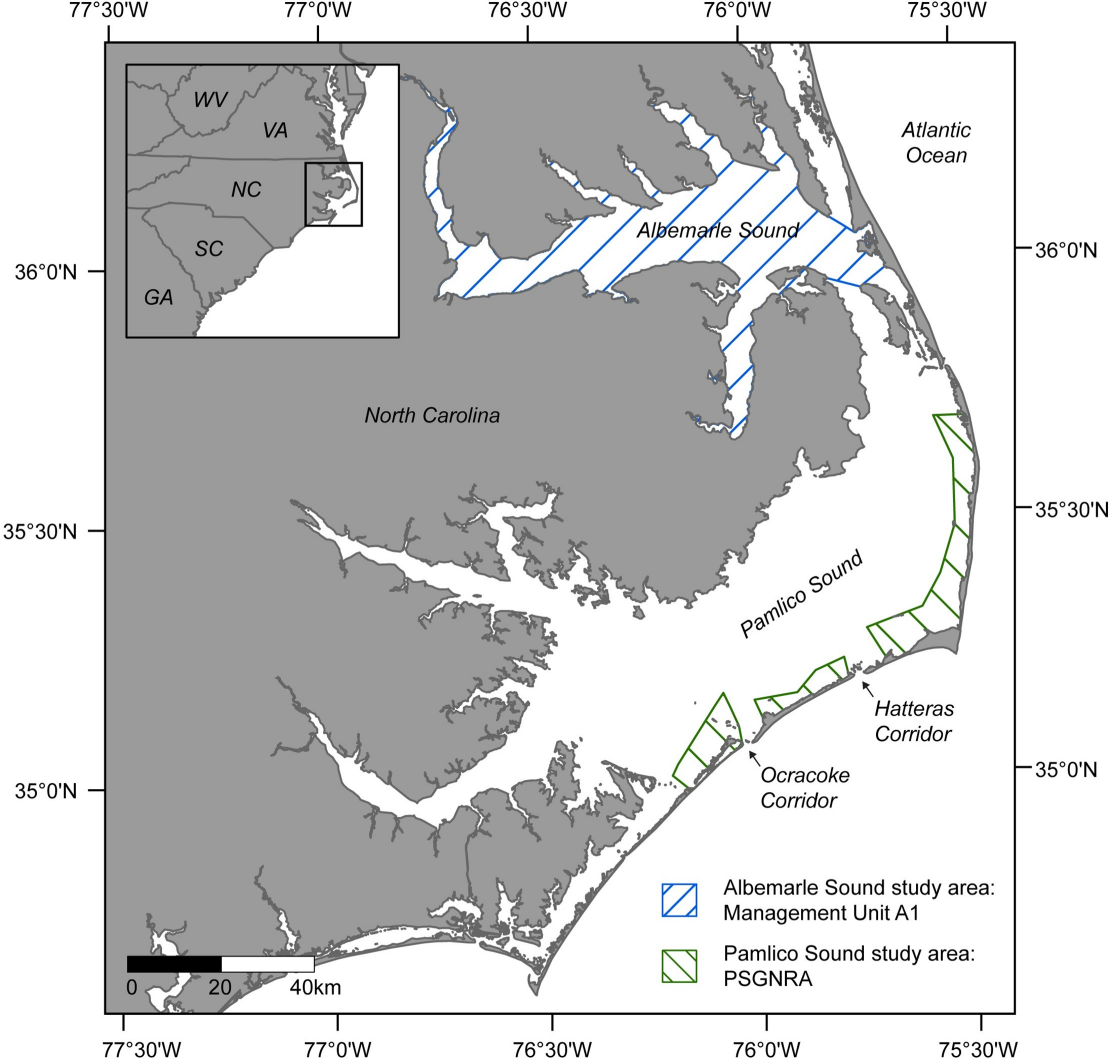
North Carolina (NC), USA coast and study areas within the Albemarle and Pamlico sounds. Both study areas comprise NC Division of Marine Fisheries management units which may be closed to gill net fishing if the number of Atlantic sturgeon or sea turtle takes approach the seasonal or annual limits specified in the Incidental Take Permit for each species. PSGNRA = Pamlico Sound Gill Net Restricted Area.

Under the current conservation management plan, if takes are approaching threshold levels, management unit A1 (which comprises the majority of Albemarle Sound) will be shut down to large mesh gill nets for the rest of the season (Fig 1). In order to avoid shutting down the fishery in this management unit entirely, NCDMF would like to delineate a smaller portion of management unit A1 that could be closed to reduce the number of Atlantic sturgeon takes.

### Sea turtle bycatch in Pamlico Sound

The majority of sea turtle takes recorded by observers in the large mesh gill net fishery in NC occur in Pamlico Sound [24]. Prior to 2000, the majority of these takes were occurring in the deep-water portions of the sound as well as in three corridors (breaks in the outer banks) that sea turtles pass through as they exit the sound during the fall months as part of their annual migration [24]. Since 2000, the deep-water portions of the Pamlico Sound as well as those three corridors have been closed to large mesh gill nets in order to reduce the number of interactions between the fishery and sea turtles [20] (Fig 1). Between 2003 and 2014, large mesh gill nets were permitted in September through December (though official start and end dates to these periods varied year to year) in some of the shallow-water portions of the sound, including the Pamlico Sound Gill Net Restricted Area (PSGNRA, Fig 1).

Even though the fishery is only permitted to operate in a small portion of the sound, managers have had to shut down the PSGNRA mid-season or delay season opening dates in past years to keep incidental sea turtle takes below the allowable threshold [20]. During spring and summer months southern flounder reside within the sound and in the fall they migrate out through the corridors to spawn offshore [27]. This migration between September and November is when the majority of southern flounder are captured by large mesh gill net fishery, and overlaps spatially and temporally with the timing of sea turtles passing through the same corridors, which leads to the increase of sea turtle bycatch during that time period [20, 27]. Managers at NCDMF would like to determine if expanding the area closed to fishing around a corridor in the PSGNRA could be an effective way of reducing sea turtle bycatch during the fall months.

## Methods

### Data

This was a data analysis and simulation study. The data analyzed in this study were from historic fisheries-independent sampling programs conducted under the auspices of the North Carolina Division of Marine Fisheries. No new field collections were done specifically for this analysis.

As part of the conservation plan implemented under the Incidental Take Permit for sea turtles, NCDMF established an observer program with 7–10% observer coverage of the large mesh gill net fishery throughout the state [24]. The program observes fishery-dependent catches; for a given catch, observers record fishing location, catch composition, time gear was fished, and characteristics of the gear used. For this study, we used all available data collected by observers in the Pamlico Sound between 2003–2014, and all available data collected by observers in the Albemarle Sound during the years 2004–2006, 2008, and 2012–2016. Due to the relatively small number of sea turtle interactions reported in the dataset, we did not distinguish between the three sea turtle species observed in the Pamlico Sound (green, Kemps ridley, and loggerhead). Fishing effort was calculated as the product of net length (yards) and the amount of time gear was fished (days) at each fishing location, as recorded by observers. Net lengths and soak times for the gear used by these fishermen averaged around 100 yards (91.4 m) and 1 day, respectively. Both float and sink gill nets were used, and mesh sizes ranged from 4.5 to 8 inches (11.4 to 20.3 cm; stretched mesh).

### Closure delineation

**Fig 2:**
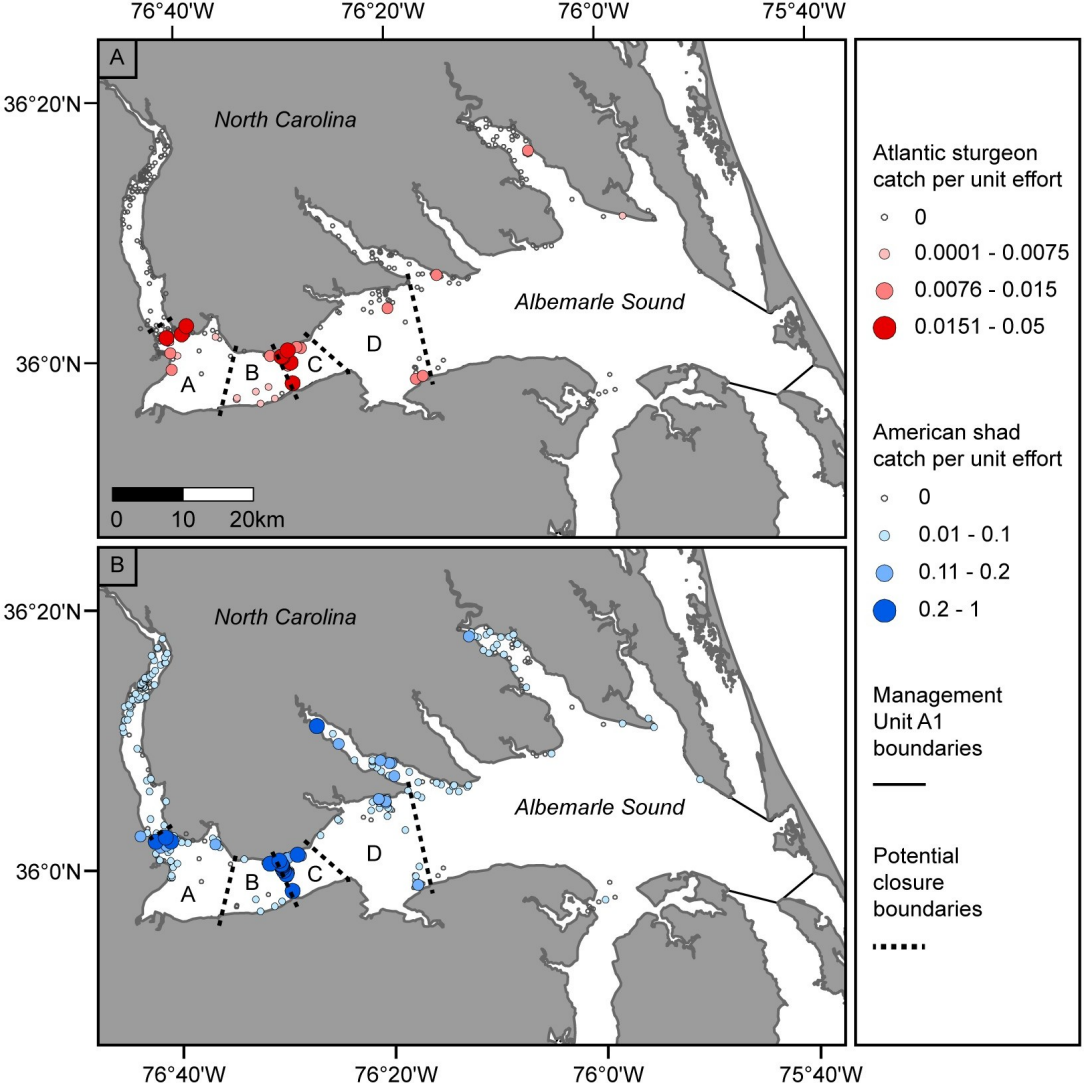
Observed catch per unit effort (CPUE) in the Albemarle Sound study area. (A) Atlantic sturgeon CPUE and (B) American shad CPUE during the months of February-April from 2004–2006, 2008, and 2012–2016. Potential closures A-D are overlaid on the map.

We plotted the catch per unit effort (CPUE) of Atlantic sturgeon and American shad in the Albemarle Sound study area for all observed nets during the American shad fishing season (February–April, Fig 2A and 2B). Bycatch of Atlantic sturgeon occurred throughout many portions of the western Albemarle Sound. We delineated four potential closures based on the location of easily enforceable geographic markers such as bridges, lighthouses, and powerlines. These closures were areas A, B, C, and D (Fig 2). We explored the effect of closing each of these areas individually on the amount of Atlantic sturgeon and American shad catch for the months of February through April.

**Fig 3:**
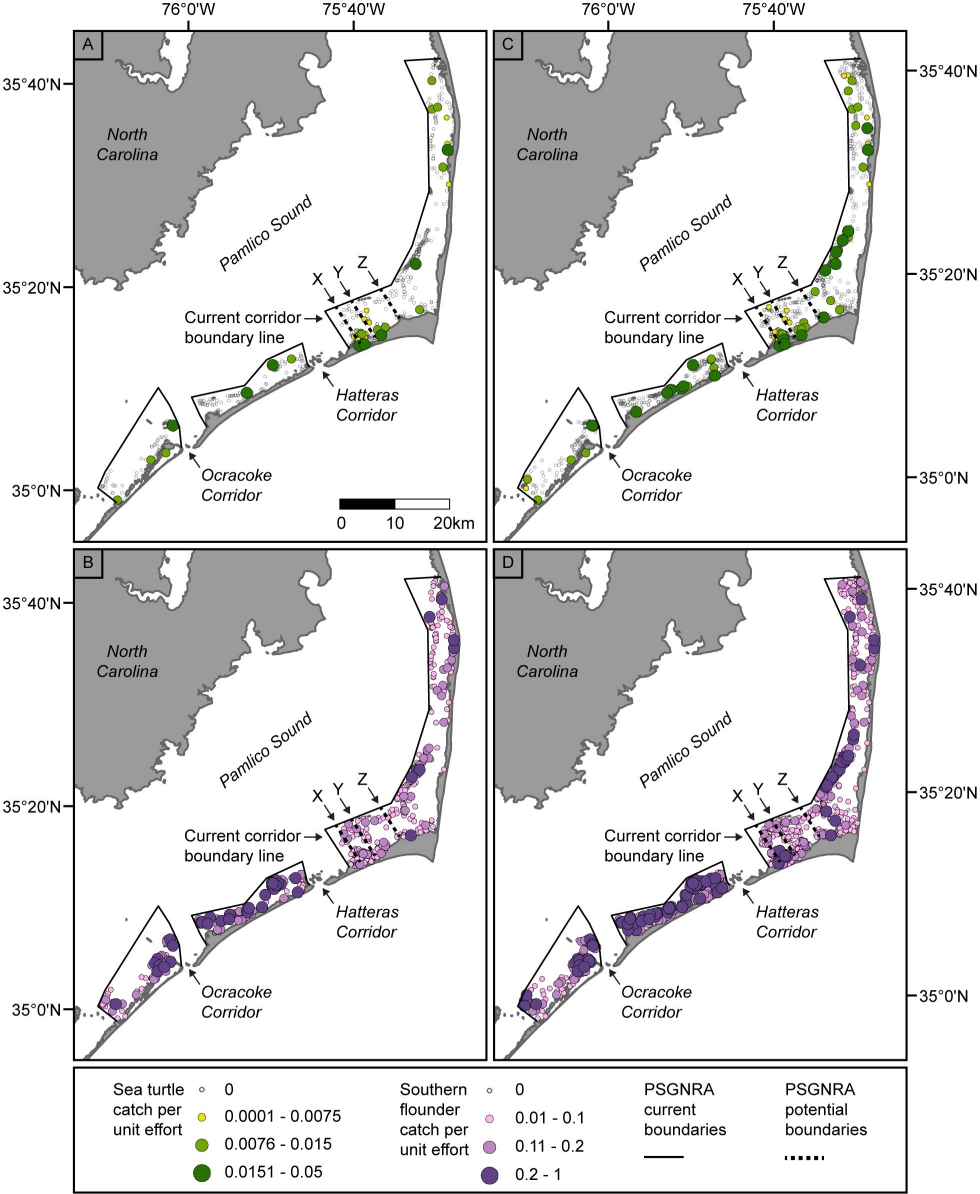
Observed catch per unit effort (CPUE) in the Pamlico Sound study area. (A) Sea turtle CPUE and (B) southern flounder CPUE during the month of September from 2003–2014, and (C) sea turtle CPUE and (D) southern flounder CPUE during the months of September-December from 2003–2014. The corridor boundary lines X-Z are overlaid on the map. PSGNRA = Pamlico Sound Gill Net Restricted Area.

We plotted the CPUE of sea turtle and southern flounder in the Pamlico Sound study area for all observed nets during the month of September alone (a time when sea turtles are abundant within Pamlico Sound, Fig 3A and 3B) and for the entire length of the southern flounder fishing season (September–December, Fig 3C and 3D). We identified a cluster of high CPUE values north of the Hatteras corridor during the month of September. The locations of three potential expanded corridor boundary lines (X, Y, and Z) were then selected based on the location of this cluster along with proximity to geographic markers such as bays, points, and islands, to increase the ease of enforceability of the putative boundaries (Fig 3). We explored the effect of expanding the corridor boundary (i.e. decreasing the size of the fished area) out to each of these lines on the amount of sea turtle and southern flounder catch. This analysis was done during just the month of September as well as for the months of September through December.

Although the use of systematic analysis to delineate spatial closures that optimize bycatch reduction and target catch maintenance would theoretically result in the most effective closures, the boundaries of these closures would be difficult to enforce because they would not coincide with landmarks that help enforcement officers and fishers determine if they within or outside of the closure. Therefore, closure boundaries were delineated by a team of biologists who work closely with enforcement officers and understand the types of landmarks that are used to demarcate and enforce closure boundaries.

For both Pamlico and Albemarle Sounds, these figures (and calculations below) use all available years of data for takes, target catch, and effort in order to provide the best predictions of closure effectiveness. It is important to note that these are cumulative across the years and not on an annual time step.

### Calculations

We estimated the impact of potential closures by calculating percent reduction in catch (%*CIC*) between the observed levels without the closure and the predicted levels if the closure had been in place for the period over which observed data were collected. We performed this calculation using the following steps:

Step 1) Total observed catch (*C*_*obs*_) and observed effort (*f*_*obs*_) for the study area were calculated as:

$$C_{obs}=C_{obs,in}+C_{obs,out}$$ (1)

$$f_{obs}=f_{obs,in}+f_{obs,out}$$ (2)

where *C*_*obs*,*in*_ and *C*_*obs*,*out*_ are the sum of observed catch across all fishing sites (observer-recorded site of net deployment) within and outside of the closure, respectively, and where *f*_*obs*,*in*_ and *f*_*obs*,*out*_ are the sum of observed effort across all fishing sites within and outside of the closure, respectively. Each fishing site corresponds to a single net deployment, i.e. there is only one observation per fishing site.

Step 2) The amount of redistributed effort (*f*_*add*_) allotted to each fishing site outside of the closure was calculated according to four possible redistribution patterns. With the exception of effort redistribution pattern 1 (in which no effort was reallocated), the sum of observed effort within the proposed closure (*f*_*obs*,*in*_) was divided among fishing sites such that the total observed effort across the study area was equal to the total predicted (*pred*) effort outside of the closure (*f*_*obs*_ = *f*_*pred*,*out*_). That is, all displaced effort was reallocated to observed fishing sites outside of the closure so that total effort did not change between the observed and predicted scenarios. Fishing sites farther than 30km from the closure were not allocated any of the redistributed effort.

Pattern 1) *No redistribution*: Displaced effort is eliminated completely. That is, all effort that occurred within the proposed closure area is removed and not reallocated to other fishing sites. The redistributed effort is calculated as:

$$f_{add}=0$$ (3)

This would be the best-case scenario in terms of bycatch reduction, but it is unlikely that all displaced effort would disappear without at least a portion of that effort shifting into areas outside the closure in this fishery. We include this pattern as a reference against which to compare the results of the other possible redistribution patterns.

Pattern 2) *Even redistribution*: Displaced effort is redistributed evenly among fishing sites within 30km of the closure boundary. The redistributed effort is calculated as:

$$f_{add}=\frac{1}{S_{obs,out<30km}}*f_{obs,in}$$ (4)

Where *S*_*obs*,*out*<30*km*_ is the number of fishing sites located outside the closure, within 30km of the closure boundary. This pattern of even effort redistribution has been used in other studies by other fishery management agencies when simulating the impacts of closures [28].

Pattern 3) *Proportional redistribution*: Displaced effort is redistributed proportionally to recorded effort at each fishing site within 30km of the closure boundary, i.e. sites with high effort are allocated a larger percentage of displaced effort. In contrast to effort redistribution patterns 1 and 2, the amount of effort redistributed to each fishing site will vary, which is denoted by the *site* subscript. The redistributed effort is calculated as:

$$f_{{add}_{site}}=\frac{f_{{obs,out<30km}_{site}}}{f_{obs,out<30km}}*f_{obs,in}$$ (5)

where $f_{{obs,out<30km}_{site}}$ is the observed effort at a given fishing site outside of the closure within 30km of the closure boundary, and *f*_*obs*,*out*<30*km*_ is the sum of observed effort across all fishing sites outside of the closure within 30km of the closure boundary. This method of redistribution is intended to simulate a situation in which fishermen redistribute to areas where they expect the target species to be in abundance, a pattern which has been observed in other fisheries [17, 18]. Here, we assume that the past distribution of fishing effort reflects local fishing knowledge about where a fisher is most likely to make a successful catch; similar approaches have been used in other studies [13, 29].

Pattern 4) *Exponential redistribution*: Displaced effort is redistributed to each fishing site within 30km of the closure boundary such that the amount decreases exponentially as the distance from the closure increases. That is, more of the effort is reallocated to fishing sites in close proximity to the closure. The redistributed effort is calculated as:

$$f_{{add}_{site}}=\frac{1/{d_{{obs,out<30km}_{site}}}^{1.5}}{\sum_{site}\left(1/{d_{{obs,out<30km}_{site}}}^{1.5}\right)}*f_{obs,in}$$ (6)

where $d_{{obs,out<30km}_{site}}$ is the distance from a fishing site within 30km of the closure to the closure boundaries. We tested multiple exponent values (greater than the typical diffusion exponent 2 and less than 2) and 1.5 produced the most realistic spatial gradient of decreasing effort with increasing distance from the closure boundary. An exponent greater than 1.5 was too extreme and less than 1.5 was too weak. This type of redistribution pattern after a closure has been observed in many different fisheries and environments [30, 31], and has often been referred to as “fishing the line” [30, 32, 33]. The behavior has been attributed to fishers attempting to minimize travel distance from old fishing grounds and fish within the expected spatial distribution of the target species [32].

Step 3) Predicted effort ($f_{{pred,out<30km}_{site}}$) at each site within 30km of the closure boundary was calculated by adding the additional effort to the original effort that was observed at each site:

$$f_{{pred,out<30km}_{site}}=f_{{add}_{site}}+f_{{obs,out<30km}_{site}}$$ (7)

Step 4) The observed CPUE for each species at each site (${CPUE}_{{obs,out<30km}_{site}}$) within 30km of the closure boundary was calculated by dividing observed catch by observed effort:

$${CPUE}_{{obs,out<30km}_{site}}=\frac{C_{{obs,out<30km}_{site}}}{f_{{obs,out<30km}_{site}}}$$ (8)

Step 5) The predicted catch for each species at each site (${C_{pred,out<30km}}_{site}$) within 30km of the closure boundary was calculated by multiplying the observed CPUE by the predicted effort:

$${C_{pred,out<30km}}_{site}={CPUE}_{{obs,out<30km}_{site}}*f_{{pred,out<30km}_{site}}$$ (9)

Step 6) The predicted %*CIC* within the study area for each closure scenario and effort redistribution pattern was calculated as:

$$\%CIC=\frac{{(C}_{pred,out<30km}+C_{obs,out>30km}){-C}_{obs}}{C_{obs}}*100$$ (10)

where *C*_*pred*,*out*<30*km*_ is the sum of the predicted catch across all fishing sites outside of the closure within 30km of the closure boundary, and *C*_*obs*,*out*>30*km*_ is the sum of observed catch across all sites greater than 30km from the closure, i.e. sites outside of the closure to which no displaced effort was allotted.

Step 7) The average %CIC for each closure scenario was calculated using a weighted arithmetic mean of the %CIC values for effort redistribution patterns 2–4. The weight of each redistribution pattern was based on relative likelihood of each pattern’s occurrence as estimated by a team of biologists familiar with the fishery. This was calculated as:

$$Average\;\%CIC=0.1*{\%CIC}_{P2}+0.3*{\%CIC}_{P3}+0.6*{\%CIC}_{P4}$$ (11)

where *P2*, *P3*, and *P4* are effort redistribution patterns 2, 3, and 4, respectively.

## Results

In both study areas, effort redistribution pattern had a large effect on bycatch and target catch levels both within and among closures. When effort redistributed into the area still open to fishing (redistribution patterns 2–4), bycatch reduction was always less than if all displaced effort was removed from the system (redistribution pattern 1). Aside from this trend, none of the other possible redistribution patterns (2–4) consistently resulted in either the highest or lowest %CIC across closures and study areas. Closures did not always lead to a catch reduction; in both study areas, some closures led to increased catch levels under certain redistribution patterns.

**Fig 4:**
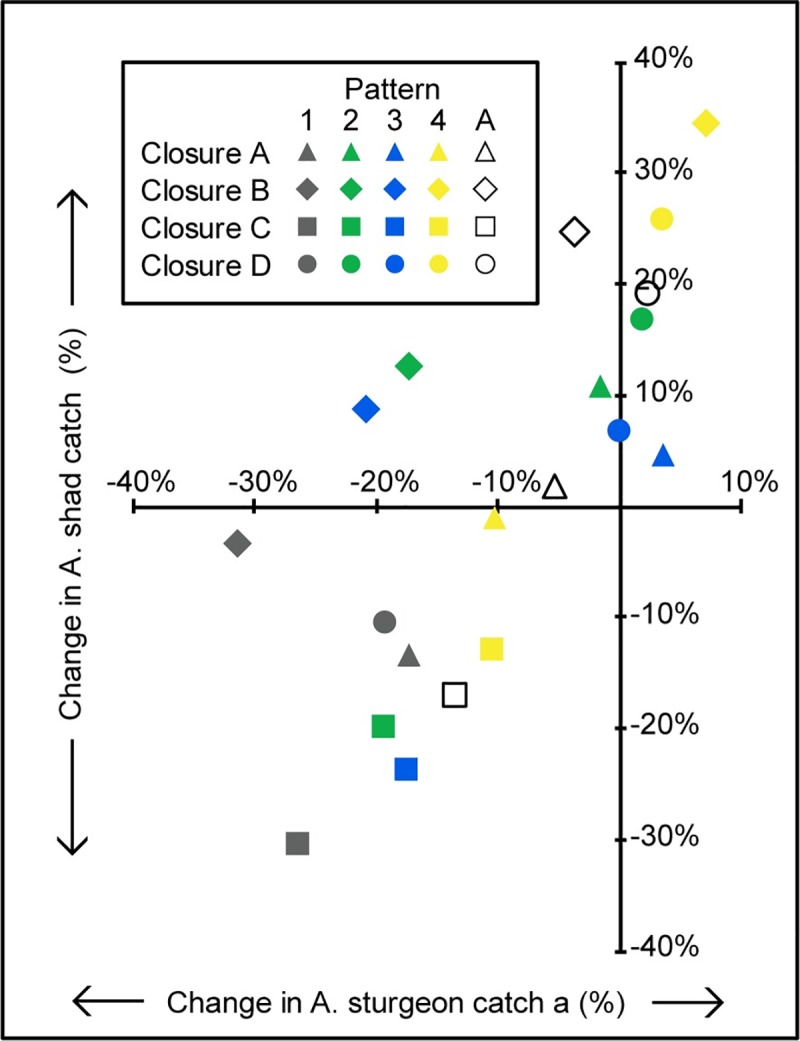
Relationship between Atlantic sturgeon and American shad change in catch (%CIC) under each combination of closure and effort redistribution pattern in the Albemarle Sound study area. Atlantic sturgeon versus American shad %CIC for all closures and possible effort redistribution patterns during the months of February-April from 2004–2006, 2008, and 2012–2016. Each combination of closure and effort redistribution pattern is represented by a color-shape combination. The four closures are each represented by a different shape; the four possible effort redistribution patterns and the weighted average of effort redistribution patterns 2–4 (A) are each represented by a different color. The possible effort redistribution patterns are 1) No redistribution, 2) Even redistribution, 3) Proportional redistribution, and 4) Exponential redistribution.

Observers reported bycatch of 57 Atlantic sturgeon in the Albemarle Sound study area during the American shad season (February–April) for the observed years (Table 1). Sturgeon bycatch was distributed throughout all four closures (Fig 2A). The %CIC of Atlantic sturgeon for each closure under redistribution pattern 1 ranged from -18 to -32% (Fig 4). The highest amount of bycatch was observed in closure B (redistribution pattern 1, Fig 4). However, this closure was only the third most effective closure at reducing bycatch (-4% average CIC) after closure C (-14% average CIC) (Fig 4). Moreover, only closure C reduced sturgeon bycatch across redistribution patterns. Closure B reduced sturgeon bycatch in three out of the four possible effort redistribution patterns, but if effort redistributed according to the fourth pattern (exponential redistribution) sturgeon bycatch increased by 7%.

**Table 1:** Observer coverage and reported Atlantic sturgeon and American shad catch in the Albemarle Sound study area during the months of February-April from 2004–2006, 2008, and 2012–2016.

Year	Observed Nets (#)	Observed Effort (yard*days)	Atlantic sturgeon (#)	American shad (#)
2004	137	17105	0	92
2005	20	2000	0	63
2006	55	31914	14	211
2008	64	9228	10	46
2013	282	26770	15	535
2014	165	17075	7	377
2015	1054	106290	9	547
2016	73	7460	2	134
*Total*	*1850*	*217842*	*57*	*2005*

American shad catch was observed in all four closures, with the largest amount observed in closure C (Fig 2B; Fig 4, redistribution pattern 1). This was the only closure that resulted in a reduction of American shad catch under redistribution patterns 2, 3, and 4. While both closures B and C led to a reduction of Atlantic sturgeon bycatch, the two closures differed in their impact on American Shad catch: closure C reduced American shad catch (-17% average CIC), while closure B increased American shad catch (25% average CIC) (Fig 4). This illustrates how time-area closures may not always have the same directional impact on both bycatch and target species catch.

**Fig 5:**
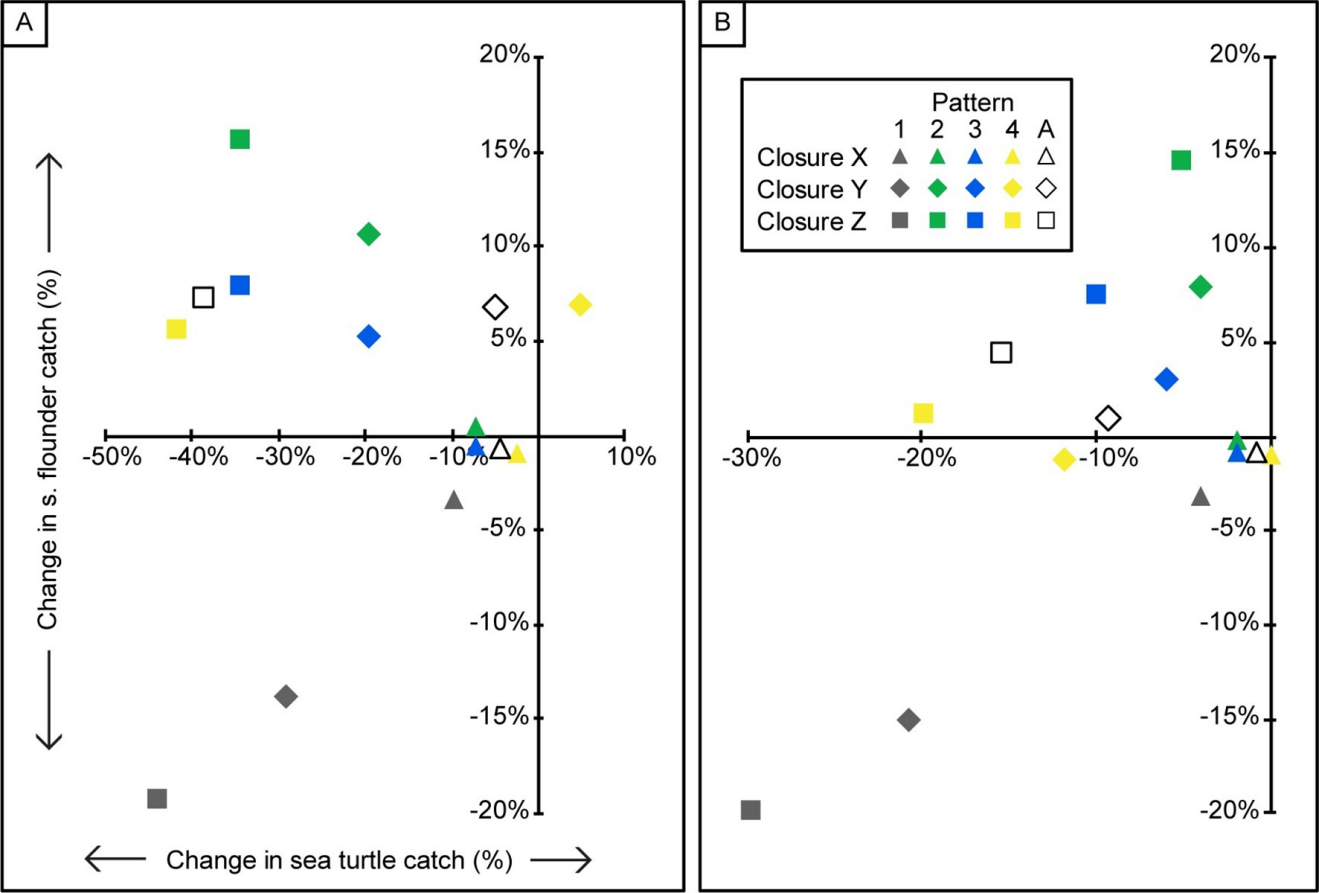
Relationship between sea turtle and southern flounder change in catch (%CIC) under each combination of closure and effort redistribution pattern in the Pamlico Sound study area. Sea turtle versus southern flounder %CIC during a) September from 2003–2012 and B) September-December from 2003–2012. Each combination of closure and effort redistribution pattern is represented by a color-shape combination. The three closures are each represented by a different shape; the four possible effort redistribution patterns and the weighted average of effort redistribution patterns 2–4 (A) are each represented by a different color. The possible effort redistribution patterns are 1) No redistribution, 2) Even redistribution, 3) Proportional redistribution, and 4) Exponential redistribution.

Observers reported bycatch of 41 sea turtles in September (Table 2), and 101 sea turtles during the entire southern flounder season (September–December; Table 3) for observed years in the PSGNRA. For both time periods, each successive expansion of the Hatteras inlet corridor (i.e. reduction of fishable area within the PSGNRA) from boundary X to Y to Z encompassed more of the observed sea turtle and flounder catch (Fig 3A and 3D). Thus, each successive expansion of the closure led to increased reductions in the sea turtle bycatch and flounder catch when displaced effort was not redistributed (Fig 5, pattern 1). Interestingly, effort redistribution (patterns 2, 3, and 4) had opposite effects on bycatch and target catch; on average, %CIC decreased and was negative for sea turtles but increased and mostly positive for flounder (i.e. increased flounder catches; Fig 5).

**Table 2:** Observer coverage and reported sea turtle and southern flounder catch in the Pamlico Sound study area during the month of September from 2003–2012.

Year	Observed Nets (#)	Observed Effort (yard*days)	Sea turtles (#)	Southern flounder (#)
2003	298	19608	0	782
2004	625	59059	3	2047
2005	540	48321	1	1979
2006	719	70622	4	1561
2007	385	36866	2	500
2008	606	63892	10	2411
2009	883	96598	19	2512
2010	444	25041	2	1409
2011	505	40243	0	1770
2012	349	29795	0	1013
*Total*	*5354*	*490046*	*41*	*15984*

**Table 3:** Observer coverage and reported sea turtle and southern flounder catch in the Pamlico Sound study area during the months of September-December from 2003–2012.

Year	Observed Nets (#)	Observed Effort (yard*days)	Sea turtles (#)	Southern flounder (#)
2003	1033	75942	4	2358
2004	1541	141267	8	4127
2005	1938	182987	4	6694
2006	2053	224930	6	6176
2007	1448	134308	19	2943
2008	1843	195563	16	5716
2009	1682	179664	33	4768
2010	2025	148435	8	6292
2011	1626	136638	0	4519
2012	888	59890	3	2647
*Total*	*16077*	*1479626*	*101*	*46240*

Counterintuitively, the month-long September closure was more effective at reducing sea turtle bycatch than the season-long closure (Fig 5). In both time periods, boundary Z was the most effective closure at reducing sea turtle takes. Over the years examined in September, there were 41 takes (Table 2) without a closure and 23 takes with boundary Z closure and no effort redistribution (pattern 1); most of the takes were within the closure area so that effort redistribution resulted in only three more takes (n = 26) and a -39% average CIC (Fig 5A). For September through December there were 101 takes (Table 3) without a closure and 71 takes with boundary Z closure and no effort redistribution. However, the relatively large number of takes once effort was redistributed (n = 89) resulted in only a -15% average CIC (Fig 5B). In other words, implementing boundary Z for the entire season was only predicted to be 80% as effective at reducing sea turtle bycatch as implementing the closure for just the first month of the season. This results from an increase in sea turtle takes to the north of boundary Z during October (after effort redistribution) which takes away from reductions in September. These results show how the timing and duration of the spatial closure can influence the amount of both bycatch and target species catch.

## Discussion

We found one or more time-area closures that are predicted to reduce bycatch under all four patterns of fishing effort redistribution in both Pamlico and Albemarle Sound fisheries. If managers were to select one of these closures, it is more likely that the bycatch thresholds would be reached later in the season, or not at all. We did not find any time-area closures that both reduced bycatch and maintained target catch at original levels. All closures were predicted to either increase or decrease target catch. Our results clearly illustrate how fisher behavior after a closure can influence levels of bycatch and target catch. Thus, managers should incorporate the potential impacts of effort displacement into closure design. Given uncertainty about fishers’ spatial response, one of the simplest and most precautionary ways to do so is to select a closure that reduces bycatch under multiple possible effort redistribution patterns.

### Effect of effort redistribution

The bycatch reduction estimates under the no redistribution pattern are likely overly optimistic unless the closure will also be accompanied by some sort of effort control [34]. Indeed, we found that the redistribution of displaced effort negatively offset the amount of bycatch reduction expected to result from the no redistribution pattern. In some cases, the closure with redistribution of effort led to higher levels of estimated bycatch than without a closure. For example, exponential redistribution of effort caused Atlantic sturgeon bycatch to increase with closure B and D and sea turtle bycatch to increase with corridor boundary Y relative to no spatial management. This pattern occurred when fishing effort was displaced into areas of high bycatch or when the closure was too small to fully encompass the bycatch cluster. Increased bycatch after a closure has been observed and predicted in other fisheries [34, 35]. For example, a study of a time-area closure in the Gulf of Maine sink gill net fishery implemented to reduce porpoise bycatch found that bycatch actually increased after the closure [34]. The authors attributed this to a combination of displaced fishing effort and the failure of the closure to fully enclose the area of highest bycatch rates due to its small size. The reluctance of fisheries managers to close off large areas to fishing has often been linked to the failure of MPAs or time-area closures to achieve their management goals [12]. Testing the effects of effort redistribution on bycatch levels could help prevent managers from choosing a closure that is too small to achieve management goals.

To address the concern of effort displacement leading to overcrowding, we performed a simple calculation for each study area. If closure X (the largest closure) in Pamlico Sound were to be closed to fishing, there would still be 270 km^2^ open to fishing within 30km of the closure. The observer coverage goal is 7–10%. If we assume 7% coverage was achieved in the year with the most observed nets (year 2015, n = 1054; Table 1), then the total number of nets in the fishery that season was approximately 15,000. Even if all of those nets were deployed only within the 30km boundary of the closure on a single day (rather than spread out over the course of the season) and each took up 100m^2^ of space, they would only require 6% of the fishable area within 30km of the closure. We performed a similar calculation for the Albemarle Sound study area, which resulted in the displaced fishers only occupying 9% of the fishable area within 30km of closure A. Of course, not all of this area would be suitable for large mesh fishers, but given that the percentage of the available space that would be required for the displaced fishers is so low, we do not believe that overcrowding would be an issue.

### Effect of closure timing

Our analysis highlighted how the timing of a closure can impact its usefulness for reducing bycatch of mobile and migratory species such as Atlantic sturgeon and sea turtles. Many would assume that longer closures would result in a greater reduction in bycatch. However, our results show that this strategy may not reduce bycatch of a mobile species when effort displacement is likely to occur. In Pamlico Sound, a “permanent” (for the entire length of the southern flounder fishing season) expansion out to boundary X, Y, and Z is predicted to be less effective at reducing seasonal sea turtle bycatch than a closure that is only in place for the first month of the season. The counterintuitive finding is a result of a spatial shift of the cluster of sea turtle bycatch to the northeast of the boundary lines during the month of October. This shift in location of bycatch along with the shift in effort caused by the closure led to an increase in sea turtle takes in October which reduces the benefits accrued in September. When designing closures to reduce bycatch of mobile species, managers should consider how the timing of the closure may enhance or detract from its ability to reduce bycatch depending on how the spatial distribution of the bycatch species varies both within and between years [34, 36, 37].

### Effect on other species

Once closure options are identified that decrease bycatch, managers can evaluate and compare them based on the predicted impact on target or other species (Figs 4 & 5). For example, if managers want to reduce bycatch while limiting the negative impact of the closure on fishers’ profits, boundary Z in Pamlico Sound is the best choice, in particular for only the month of September (Fig 5A). This closure is predicted to both increase flounder catch and decrease sea turtle bycatch under effort redistribution patterns 2–4. This occurs because fishing effort is displaced into areas where observed southern flounder CPUE was higher and observed sea turtle CPUE was lower than inside the closure. A study in the North Sea that simulated the effects of potential fishing closures also predicted an increase in target catch resulting from effort displacement [29]. One critical assumption for the increase in target catch is that CPUEs would be maintained after extra effort moves in. This raises the question of why some areas with high catch rates of the target species would have low effort. Possible explanations include the fact that fishers don’t have complete and perfect information on target species location and likely don’t share the geographic location of highly productive spots they have discovered. Even if high CPUE areas are known to fishers, the distance to these sites could exclude the majority of fishers. Additionally, fishers may be loyal to certain sites and may not frequently test new areas.

The choice of best closure will differ depending on management goals. It may not always be desirable to increase catch of the target species, for example, if management is in place to maintain or decrease existing catch levels. Closure C in Albemarle Sound is predicted to be the most successful at reducing Atlantic sturgeon bycatch, but leads to an average decrease in American Shad catch (Fig 4). Although on average closure A is less effective at reducing Atlantic sturgeon bycatch, it is predicted to have a less detrimental effect on fishers’ catch of shad and therefore earnings. Here managers would have to decide whether to choose the closure with higher average predicted bycatch reduction in sturgeon at the cost of shad catch (closure C) or the closure with lower average predicted bycatch reduction that is less likely to negatively impact fishers’ profits from American shad (closure A). In this way managers can explicitly consider the impacts of a closure on both bycatch and target species and decide how each closure aligns with their management goals. Similarly, Watson et al. [38] calculated a catch to bycatch ratio from past catch data to evaluate potential spatial closures in the eastern Pacific Ocean tuna fishery on their ability to minimize the tradeoff between foregone tuna catch and reductions in bycatch of silky shark. Although their study did not consider how effort reallocation after the closures would impact their results, they did identify this as an important direction of future study and as a major source of uncertainty when choosing policies that must balance impacts on a variety of species [38].

### Caveats to our analysis

We made simplifying assumptions in our analysis to increase the ease and speed of implementation and interpretation. First, we assumed that all displaced effort redistributed throughout areas still open to fishing (up to 30km) in redistribution patterns 2 to 4. This is likely unrealistic as some fishers may exit the fishery when displaced from their preferred fishing grounds. Thus, the offset of redistribution patterns 2 to 4 relative to pattern 1 may not be as severe. Second, we assumed that effort only redistributes spatially, not temporally. In reality, if a fisher’s primary fishing grounds are closed for a portion of the season, that fisher may simply delay fishing until that area reopens. Third, we assumed that CPUE before and after the closure would remain the same. Catch rates of target species could decrease as a result of increased effort (same effort over smaller area or increased effort per fisher to make up for higher travel costs) and decreased densities of the target species. For this reason, predicted increases in catch of the target and bycatch species may be overinflated, which is a common caveat to these types of analyses [13, 18, 29]. Alternatively, CPUE of the target species could increase if spatial closures resulted in further travel costs and fishers elected to drop out of the fishery leading to less competition for prime fishing spots. Future work should examine for a relationship between catch and effort to determine if the assumption of fixed CPUE is valid. Lastly, we assumed that the location and timing of historical bycatch and target catch over many years are representative of future catches.

We used redistribution patterns of displaced effort that we believed were realistic for this fishery but there are other patterns that could be explored in future work. For example, a pattern that redistributes displaced effort in proportion to historic catch rates of target species would set an upper bound on target catch. Similarly, a lower bound on bycatch would be found using a pattern where effort was redistributed in inverse proportion to historic catch rates of bycatch. Lastly, the effect of travel costs of displaced effort could be incorporated into a redistribution pattern where effort is inversely proportional to travel distance from ports to open fishing areas.

There are limitations to the dataset that may have affected our results. The observed coverage of these two fisheries represented 7–10% of trips made by the fishery. We assumed that this is a representative sample of the fishery, but it is possible that this was not enough coverage to capture the true spatial distribution and magnitude of catch and effort. Although the dataset spans many years, this low coverage rate also means that the dataset itself is relatively small. Using the methods outlined in this paper with a small dataset and therefore a small number of recorded instances of bycatch means that one observation could play a large role in the projected bycatch/catch rates. Lastly, commercial fishermen with observers on board may have changed their fishing behavior and spatial strategies in the presence of observers, therefore misrepresenting and potentially obscuring true bycatch and target catch rates.

In this modeling exercise we only reallocated displaced effort to existing fishing sites within 30km of the closure boundaries (see Methods). This redistribution distance was chosen based on conversations with fisheries managers about their predictions of how far gill net fishers would be willing to travel if displaced from their preferred fishing grounds. However, displaced effort might move less or more than 30km. In addition to the presented results above, we also tested the redistribution distances of 15km, 45km, and no maximum distance within the boundaries of the study area. The overall results did not change when varying the redistribution distance. Although not important in our study, we urge others to test if redistribution distance has an effect in their particular study system.

The results of this type of modeling exercise should only be used to evaluate potential temporary or seasonal closures that are evaluated annually and are not intended to be used to design permanent closures or marine reserves. Once closures are implemented, we recommend careful monitoring of catch and effort displacement to test model predictions and ensure that management goals are being met. New data should be re-incorporated into the models and the placement of closed areas should be updated accordingly in subsequent years or iterations.

In addition to these monthly and seasonal time-area closures, we recommend investigating the potential for even shorter time-scale, dynamic spatial management to be applied in this (or similar) systems [39]. These methods would not rely as heavily on some of the major concerns listed above: historic CPUE data and effort redistribution scenarios. The observed trips in North Carolina could provide real time information on bycatch and be used to implement “move-on” rules where fishers would not fish bycatch hot spot areas for a set period of time. This would avoid the fixed time-area closures that might miss bycatch hot spots that vary from year to year or are nullified by movement of effort into another hot spot area. For example, Dunn et al (2014) used spatiotemporal autocorrelation analyses to determine the distance to move and time to stay away from bycatch hot spots in the New England multispecies fishery [40].

### Ease of use

It is widely recognized that effort redistribution can determine the success or failure of area closures to achieve bycatch reduction or other conservation goals [12, 14, 16, 19]. However, most of the techniques presented in the literature that would allow fisheries managers to model how effort redistribution may affect their goals are both data- and time- intensive. Techniques include the DISPLACE model [41], Honeycomb model [42], Ideal Free Distribution and fleet dynamics models [14], and random utility models [13]. Simulating possible redistribution patterns using these types of models may prove impractical or infeasible for many regional agencies with limited time and funding, so the straightforward calculations outlined in this paper may be more useful to managers. There have been many studies that have used calculations similar to ours to estimate patterns of effort redistribution (though often in conjunction with more complex modeling approaches) [13, 14, 28] and numerous other studies that have recognized the importance of designing closures that are expected to achieve management goals in the face of uncertainty [43]. However, we are not aware of other studies that used a relatively simple approach to identify closures that are that are effective under multiple spatial patterns of fishing effort displacement in an estuarine gill net fishery.

Differences between the predictions of models used to inform fisheries management decisions and reality are inevitable, though ideally the differences are small enough that management strategies based on these predictions are still effective. There are few studies that have compared predicted and observed outcomes of closures [44]. Valcic [45] simulated how effort would redistribute after a closure for 15 study areas and found their predictions did not match empirical observations in four out of fifteen study areas. These differences between predictions and reality were caused by a shift in the way fishers selected suitable fishing sites after the introduction of the closed area. The authors concluded that the differences were large enough that any management strategies devised based on this simulation may have resulted in unintended consequences, or would not appropriately address the problems that arose from effort displacement. This example shows that even when rigorous statistical and economic modeling is being performed, the systems being modeled are so complex that key assumptions may be wrong or could change, causing predictions to fall short of reality [19, 43, 46]. In general, designing management strategies that are independent of assumptions about human behavior and effective under multiple spatial patterns of effort redistribution will increase the likelihood of achieving management goals.

## Conclusion

The methodology outlined in this paper will aid managers in evaluating potential time-area closures without the burden of complex and data-intensive modeling tasks. The approach also provides a concrete way for managers to weigh the gains of expected bycatch reduction against the perceived costs of lost target species catch due to banning fishing from historical fishing grounds. The results have already been useful in helping managers incorporate fishing effort displacement into their decision-making process when selecting closures to reduce bycatch in NC. For example, the closures X, Y, and Z were considered for the 2016 fishing season. Although this approach does not guarantee bycatch reduction with no significant impact on the fishery, it does bring to light many of the issues that managers need to consider in closure design such as effort redistribution, closure timing, placement of closures, and effects on target catch.
